# First and Second Line Chemotherapeutic Regimens for Non-Small Cell Lung Carcinomas - The Efficacy of Platinum, Non-Platinum and Combination Therapy: A Literature Review

**DOI:** 10.7759/cureus.11619

**Published:** 2020-11-22

**Authors:** Anita Michael, Alaine Ainsley, Alan Joseph, Nusrat Jahan

**Affiliations:** 1 Internal Medicine, California Institute of Behavioral Neurosciences & Psychology, Fairfield, USA; 2 Cardiology, Rush Medical Center, Chicago, USA; 3 Cardiology, California Institute of Behavioral Neurosciences & Psychology, Fairfield, USA

**Keywords:** nsclc, chemotherapy, platinum based, docetaxel, gemcitabine, combination therapy

## Abstract

Non-small cell lung carcinomas (NSCLC) account for a major part of all lung cancer diagnoses. The current literature review is aimed to analyze the varied chemotherapeutic treatment regimens available and to provide a standard for their use in the present and future scenarios. The current literature review focuses on platinum, non-platinum and combination therapeutic modalities, in the first and second line setting. The review also ensures that docetaxel and/or gemcitabine is a part of the study. A PubMed search for NSCLC identified 70,077 articles. A total of 36 research articles were obtained following the application of keywords and inclusion/exclusion criteria to narrow down our search to meet with the research objective. These articles consider NSCLC and chemotherapeutic treatment modalities as its primary endpoint. These 36 articles included 15 randomized clinical trials, five randomized control trials, five retrospective cohort studies, one case-control study, six review articles and four observational studies. Our analysis shows that there is an increasing potential for the use of non-platinum based drugs in the clinical setting with an efficacy that is at par with that of platinum-based treatment modalities. In fact, the studies have proven a greater advantage with the use of combination therapy (non-platinum + platinum), which can be readily applied as an alternative in the clinical setting while the use of non-platinum drugs (other than docetaxel) as a monotherapy or in combination with other non-platinum based drugs does require further research.

## Introduction and background

Non-small cell lung carcinomas (NSCLC) account for about 80-85% of all lung cancer diagnoses [1]. The main sub-types are adenocarcinoma, squamous cell carcinoma and large cell carcinomas. These carcinomas arise from different types of lung cells but are grouped together as NSCLC. This is because they share a similar prognosis and treatment [2]. Most stage one and stage two NSCLC are treated by surgical resection of the tumor followed by chemotherapeutic regimens [3]. However, the initial signs are often ignored and most patients present with advanced-stage NSCLC, which are non-operable at the time of diagnosis. Although the incidence of mortality is high, especially in the case of advanced NSCLC, chemotherapeutic drugs have proved effective in increasing the overall survival (OS) rate [1]. An improvement in the median survival time (MST) by about one and a half to three months can be observed in such individuals [4]. The conventional treatment regimens involve platinum-based chemotherapy. European guidelines recommend a cisplatin-based therapy while American guidelines largely advocate for a platinum-based doublet [5]. Cisplatin is preferred to carboplatin because of better effect on survival as compared to other platinum-based regimens [5]. The last one to two decades saw the advent of non-platinum based regimens involving drugs like docetaxel and gemcitabine into the market which were seen as potential alternatives for the traditional platinum-based therapy. At present, non-platinum based regimens are administered only if platinum-based regimens are contraindicated. Docetaxel is seen as the gold-standard for second-line treatment [5] while gemcitabine is slowly accepted as a first-line drug when given in combination with cisplatin [6]. While platinum-based therapy is associated with an increased risk of toxicity, it is imperative to come up with newer treatment regimens that can replace or reduce this risk of toxicity while proving to be equally efficient in terms of overall survival.

In the presented literature review, the drugs used under the European and American guidelines (carboplatin and cisplatin) will be considered under platinum-based regimens. To narrow down our study, all non-platinum based regimens will be considered, with inclusion criteria such that docetaxel and gemcitabine are a part of the research study. Docetaxel and gemcitabine are the most commonly used drugs as a part of the non-platinum treatment regimen. Our aim is to compare the efficiency and uses of platinum-based, non-platinum based, and combination therapy in the treatment of NSCLC. The current literature review hopes to provide greater insight into the available combinations while also giving a standard to compare and administer the appropriate first- and second-line treatments for NSCLC.

## Review

Method

Literature was searched in the PubMed database. Parallel strategies on ‘regular keywords’ and ‘Medical Subject Heading (MeSH) subheadings’ were used for data collection. No other database was taken into consideration. Table 1 below further illustrates the above methodology.

**Table 1:** Literature Search using Regular and Medical Subject Heading (MeSH) Subheadings NSCLC: Non-Small Cell Lung Carcinomas

Regular Keyword – NSCLC	
Total Records	70,077
Records Selected	26,405
Regular Keyword NSCLC; Chemotherapy	
Total Records	32,117
Records Selected	12,599
Regular Keyword NSCLC; Chemotherapy Platinum based	
Total Records	2646
Records Selected	1262
Regular Keyword NSCLC; Chemotherapy Platinum based docetaxel and gemcitabine	
Total Records	198
Records Selected	97
MeSH Keyword Carcinoma, non-small cell lung Subheading: Drug Therapy	
Total Records	20,339
Records Selected	8879

The articles collected from PubMed search results were filtered as per the criteria below:

Inclusion Criteria

Only English language articles with full-text availability were considered. All of these articles were published in Medline journals and were of the following study types: observational studies, cohort studies, case-control studies, review articles, and clinical trials including randomized clinical trials. These studies/review articles were based on first- and second-line chemotherapeutic regimens and involved platinum/non-platinum based therapies like docetaxel and gemcitabine. These studies were based on an adult population (age 19+) as a reference point.

Exclusion Criteria

Non-English language literature and those studies that have not been published in Medline were not considered as a part of the review. All studies conducted in the laboratory on an animal model and the study types involving meta-analysis, case reports and case-series studies have been excluded. Studies/trials conducted on pediatric age groups and on individuals with two or more chemotherapeutic drug therapies were further excluded from the review.

Review

Table 2 shows the total number of literatures following the application of each inclusion/exclusion criteria to regular and MeSH keywords.

**Table 2:** Total number of literatures following application of each inclusion/exclusion criteria. NSCLC: Non-Small Cell Lung Carcinomas, MeSH: Medical Subject Heading

Regular Keyword – NSCLC	
Total Records	70,077
Inclusion/Exclusion	
Medline Journal	59,516
English Language	54,317
Humans	53,849
Adults 19+	28,204
Full Text	26,405
Regular Keyword NSCLC; Chemotherapy	
Total Records	32,119
Inclusion/Exclusion	
Medline Journal	29,242
English Language	26,495
Humans	26,300
Adults 19+	13,629
Full Text	12,599
Regular Keyword NSCLC; Chemotherapy Platinum based	
Total Records	2646
Inclusion/Exclusion	
Medline Journal	2238
English Language	2093
Humans	2089
Adults 19+	1299
Full Text	1262
Regular Keyword NSCLC; Chemotherapy Platinum based docetaxel and gemcitabine	
Total Records	198
Inclusion/Exclusion	
Medline Journal	187
English Language	170
Humans	170
Adults 19+	99
Full Text	97
MeSH Keyword Carcinoma, non-small cell lung Subheading: Drug Therapy	
Total Records	20,339
Inclusion/Exclusion	
Medline Journal	20,339
English Language	18,520
Humans	18,418
Adults 19+	9,576
Full Text	8,879

The selected 12,599 articles described chemotherapeutic drug treatment of NSCLC. A total of 12,502 articles were removed due to lack of specificity towards platinum and non-platinum based treatment regimens. On a refined search, 97 full articles were obtained. All of the 97 articles were reviewed and 61 articles were removed. These 61 articles correspond to: two meta-analysis reports, one duplicate/repeated article, two articles with trials that stopped midway or were not possible to extract data, and 56 articles which failed to comply with our interest towards chemotherapeutic treatment regimens (studies that compared chemotherapy along with radiation therapy, compared toxicities without emphasis over efficacy, genetic polymorphisms related to NSCLC, etc.).

Therefore, 36 articles were reviewed in PubMed and these included: 15 randomized clinical trials, five randomized control trials, five cohort studies (all retrospective cohort studies and no prospective cohort studies), four observational studies, one case control study and six review articles. The maximum number of subjects in a study was 13,472 and the minimum was 27 individuals.

A Brief Analysis of Results 

The articles analyzed the efficacy of platinum, non-platinum or combination therapeutic regimens while also advocating towards specific treatment modalities. Nine studies advocate for combination therapy as an effective treatment regimen for first-line NSCLC [7-15], five studies advocate towards non-platinum based therapy as an effective and safer regimen [8,16-19], one study concludes that non-platinum based therapy is an effective regimen compared to combination therapy in first-line NSCLC [20], two studies conclude that both non-platinum based regimens and combination therapy show an equal efficacy [21,22], three therapies indicate similar efficacy between platinum-based and non-platinum based regimens [23-25], two therapies indicate that the efficacy of non-platinum based therapy is similar to the efficacy of other treatment regimens [26,27], one study states that platinum-based regimens showed a higher efficacy and response rate than non-platinum based regimens [28], 12 studies advocate for non-platinum based regimens as a second line treatment regimen [29-40], and two studies advocate towards combination therapy for second-line treatment [41,42]. Most of the articles lean towards the use of combination therapy for first-line treatment and non-platinum based therapy for second-line treatment. Among non-platinum based therapy, studies mostly advocate for the use of docetaxel or the combination of docetaxel with other non-platinum drug therapies.

**Figure 1:**
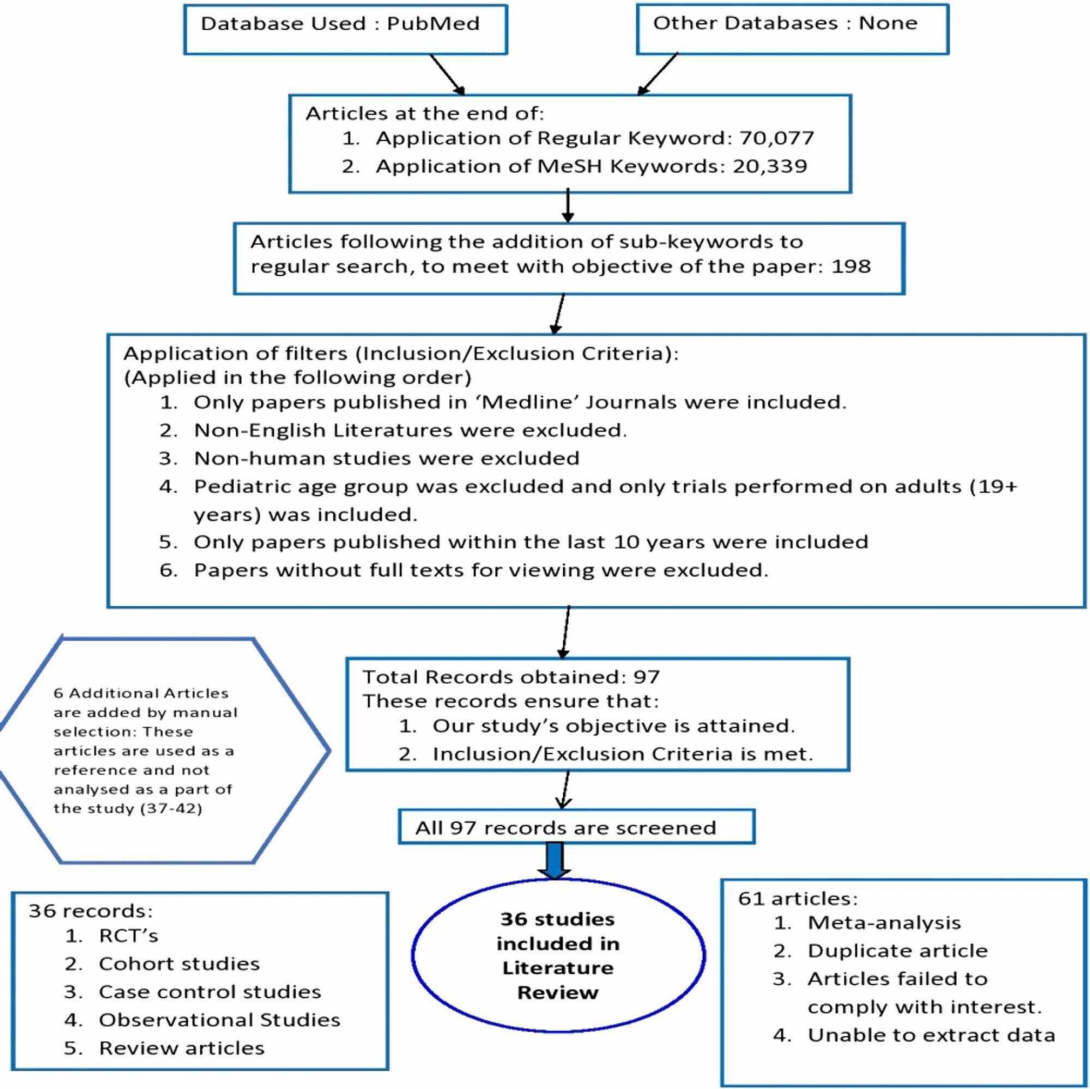
Procedure of Literature Review RCT: randomized controlled trial

The statements used in this article have been cited from the corresponding journals and the references are available. The available articles following inclusion/exclusion criteria have also been subjected to a qualitative review so as to ensure that the relevant disease, the chemotherapeutic treatment regimens, population and the outcome can be studied. A summary of the methods and results is given in Figure 1 below.

Discussion

The analysis mainly recognizes the use of non-platinum treatment regimens like docetaxel and gemcitabine or at the least, to incorporate them along with traditional platinum-based modalities of treatment. Platinum-based modalities are associated with an increased risk of toxicity which is imperative to study the use of non-platinum based regimens to eradicate or decrease the toxicities with long term administration. In this article, the efficacy of these regimens as first-line and second-line drugs have been evaluated. A good mean survival time (MST) is seen in both the first- and second-line setting with the use of these regimens. Some of the studies that describe the efficacy of non-platinum-based and combination therapy as first-line treatment regimens are described in Table 3.

**Table 3:** Summary of studies involving first-line drug regimens in non-small cell lung carcinomas (NSCLC). MST: Median Survival Time; Duration is expressed as months. ORR: Overall Response Rate; Expressed in Percentage (%).

Author/ YOP	Study Design	Population Considered	Sample Size	Main points	MST*
Marisa A Bittoni, et al. 2018 [7]	Retrospective Cohort Study	Stage IIIB/IV NSCLC patients considered. 46% of patients were given systemic therapy and 5931 patients were given platinum-based therapy.	13,472	Combination therapy (doublets with carboplatin) were mostly used with the frequency of use following the following order: carboplatin/paclitaxel>carboplatin/pemetrexed>carboplatin/gemcitabine>carboplatin/docetaxel.	7.2
John D Hainsworth,et al. 2003 [38]	Randomized Clinical Trial	Advanced NSCLC patients with poor performance status (PS). Median age of Population was 70 years.	64	Up to 30% survived at one year, 17% at 2 years and 28% showed an objective response to treatment with the IV administration of docetaxel and gemcitabine on days 1,8 and 15.	7
Marcus A Neubauer, et al. 2005 [35]	Randomized Control Trial	Advanced NSCLC patients considered. Median age of population was 68.5	50	Up to 32% survived at one year and a median disease free progression of 5.1 months shows efficacy of weekly docetaxel administration with Gemcitabine at days 1,2,4 and 5.	6.9
Domenico Galetta, et al. 2002 [39]	Observational Study	Stage IIIB/IV NSCLC patients considered. Performance status (PS) of 0-2. Median age of population was 65 years.	47	Up to 38% survived at one year with a median duration of response of six months when bi-weekly combination of docetaxel and gemcitabine is given.	10.5
Sujith Kalmadi, et al. 2007 [23]	Randomized Control Trial	Advanced NSCLC patients with 38 at Stage IV disease. Performance status (PS) of 0-1. Median age of Population was 63 years.	42	Up to 48% survived at one year and a median disease free progression of 5.1 months shows efficacy of docetaxel and gemcitabine at weekly administration.	11.3
Irina E Popa, et al. 2002 [19]	Randomized Clinical Trial	Advanced NSCLC patients considered.	32	Up to 35% survived at one year, 19% at two years with an Overall response rate (ORR) of 30% when docetaxel and gemcitabine are given on days 1 and 8 in a 21 day cycle.	7.9

Docetaxel and gemcitabine show up to two years survival rates in patients with advanced NSCLC [38] and an MST of up to 11.3 months in the studies evaluated [23]. These statistical evidences provide encouragement towards the use of non-platinum modalities in the treatment of NSCLC, especially advanced NSCLC. Some of the studies that describe the efficacy of non-platinum-based and combination therapy as second-line treatment regimens are described in Table 4.

**Table 4:** Summary of studies involving second-line drug regimens in non-small cell lung carcinomas (NSCLC). MST: Median Survival Time; Duration is expressed as months. ORR: Overall Response Rate; Expressed in Percentage (%).

Author/ YOP	Study Design	Population Considered		Sample Size	Main Points	MST*
N Kentepozidis, et al. 2017 [20]	Randomized Clinical Study		Patients presenting with advanced NSCLC. Patients are pretreated with docetaxel/gemcitabine.	124	An ORR (Overall Response rate) of 18% was seen with irinotecan on days (1 and 8) + cisplatin (REG A) given on day 8 and an ORR of 19% was seen with pemetrexed/cisplatin given on day 1. (REG B)	REG A = 6.9 REG B = 10.9
Fatma Yildrim, et al. 2015 [21]	Retrospective Cohort Study		Patients presenting with advanced NSCLC. Patient should have undergone 1 prior platinum-based chemotherapy.	57	40 patients were treated with docetaxel(REG A) and 17 with gemcitabine (REG B) monotherapy to achieve an Overall Response rate (ORR) of 12% for docetaxel and 8% for gemcitabine.	REG A = 21 REG B =22
Manuel Cobo, et al. 2007 [34]	Observational Study		Patients presenting with Advanced NSCLC and previously treated with platinum-based regimens. Performance status of 0-1.	52	An overall response rate of 28% was achieved with an administration of docetaxel and gemcitabine IV on days 1 and 8.	8.2
C H Spiridonidis, et al. 2001 [40]	Randomized Clinical Study		Patients presenting with Advanced NSCLC. Prior chemotherapy involves platinum based regimens in 36 patients, vinorelbine in 26 patients and etoposide in 10 patients.	40	A median time to progression of nine months seen with an administration of monthly docetaxel and weekly gemcitabine.	8.1
Fumiyoshi Ohyanagi, et al. 2006 [24]	Randomized Clinical Study		Patients presenting with Advanced NSCLC. 21 patients were previously treated with platinum+taxanes, 17 patients with carboplatin+paclitaxel and 4 patients with cisplatin/carboplatin+docetaxel.	27	Up to 34.8% survived up to one year with a gemcitabine and irinotecan combination administered on days 1 and 15.	7.7
F Nelli, et al. 2004 [26]	Randomized Clinical Study		Patients presenting with Advanced NSCLC. Patients should have undergone prior combination chemotherapy involving cisplatin/gemcitabine or carboplatin/paclitaxel.	27	Up to 30% survive at 1 year on administration of weekly docetaxel with vinorelbine regimens.	8

The MST of docetaxel and gemcitabine are 21 and 22 months, respectively, when used as monotherapies [21]. Docetaxel, though considered to be a gold-standard for the second-line treatment of NSCLC, gemcitabine shows equal potential that can be studied further as an alternative to docetaxel.

Overall, in terms of first-line treatment, we found nine articles that advocate for combination therapy and they comprise the following study designs: retrospective cohort study [7], randomized clinical trial [8], randomized control trial [10], case control study [11], randomized clinical trial [12], randomized control trial [13], observational study [14], review article [15]; five articles advocate for non-platinum regimens and they comprise the following study designs: randomized clinical trial [8], review article [16], observational study [17], randomized clinical trial [18], randomized clinical trial [19]; one article (retrospective cohort study [28]) advocates for the use of non-platinum based therapy over combination therapy.

In terms of second-line treatment, 12 articles advocate for the use of non-platinum regimens and they comprise the following study designs: randomized clinical trial [29], randomized clinical trial [30], retrospective cohort study [31], retrospective cohort study [32], randomized clinical trial [33], observational study [34], randomized control trial [35], review article [36], randomized clinical trial [37], randomized clinical trial [38], observational study [39], randomized clinical trial [40] and two studies (randomized clinical trial [41], randomized control trial [42]) advocate for the use of combination therapy. On the basis of the articles analyzed, we find an increasing number of articles that advocate towards the use of non-platinum and combination chemotherapeutic modalities. 

Limitations of the Review Article

Despite the analysis of efficacy between therapeutic regimens, individual drugs within the non-platinum therapeutic schemes weren’t analyzed separately. The standard drugs, docetaxel and gemcitabine, were used to drive the research forward with little emphasis over the other non-platinum based drugs and their efficacies. An equal number of studies involving different stages of NSCLC and the efficacy of these drugs in each of those stages were impossible to extract with a vast majority of studies leaning towards advanced stage NSCLC’s, possibly owing to the lack of diagnosis at the early stages with this disease. Every study was unique in its manner of administration of the same drug, with differences in the dosage/cycles/period of treatment with that specific drug. The accurate dosage or the changes in efficacy and responses with the dosage could not be evaluated in this study. Nevertheless, we have tried to obtain a standard to consider and re-evaluate the uses of newer drug combinations with varied efficacies.

## Conclusions

On the basis of the study results, we can conclude that the current literature review aligns with our research objective to compare the efficiency and the uses of platinum and non-platinum treatment regimens. We can thus conclude that there is an increasing potential for the use of non-platinum based drugs in the clinical setting with an efficacy that is at par with that of platinum based treatment modalities. In fact, the studies have proven a greater advantage with the use of combination therapy (non-platinum + platinum), which can be readily applied as an alternative in the clinical setting. However, as for the use of other non-platinum drugs as a monotherapy or along with other non-platinum drugs, other than that of docetaxel (used in second-line therapy), does require further research over its efficacy and advantages over conventional treatment modalities.
